# Marker-Based Multi-Sensor Fusion Indoor Localization System for Micro Air Vehicles

**DOI:** 10.3390/s18061706

**Published:** 2018-05-25

**Authors:** Boyang Xing, Quanmin Zhu, Feng Pan, Xiaoxue Feng

**Affiliations:** 1 School of Automation, Beijing Institute of Technology, Beijing 100081, China; golaced@163.com (B.X.); panfeng@bit.edu.cn (F.P.); 2 Faculty of Computing, Engineering and Mathematical Sciences, University of the West of England, Bristol BS16 1QY, UK; quan.zhu@uwe.ac.uk; 3 Kunming-BIT Industry Technology Research Institute INC, Kunming 650106, China

**Keywords:** indoor localization, ArUco marker, federated filter, Micro Aerial Vehicle

## Abstract

A novel multi-sensor fusion indoor localization algorithm based on ArUco marker is designed in this paper. The proposed ArUco mapping algorithm can build and correct the map of markers online with Grubbs criterion and K-mean clustering, which avoids the map distortion due to lack of correction. Based on the conception of multi-sensor information fusion, the federated Kalman filter is utilized to synthesize the multi-source information from markers, optical flow, ultrasonic and the inertial sensor, which can obtain a continuous localization result and effectively reduce the position drift due to the long-term loss of markers in pure marker localization. The proposed algorithm can be easily implemented in a hardware of one Raspberry Pi Zero and two STM32 micro controllers produced by STMicroelectronics (Geneva, Switzerland). Thus, a small-size and low-cost marker-based localization system is presented. The experimental results show that the speed estimation result of the proposed system is better than Px4flow, and it has the centimeter accuracy of mapping and positioning. The presented system not only gives satisfying localization precision, but also has the potential to expand other sensors (such as visual odometry, ultra wideband (UWB) beacon and lidar) to further improve the localization performance. The proposed system can be reliably employed in Micro Aerial Vehicle (MAV) visual localization and robotics control.

## 1. Introduction

Global position system (GPS) is the main localization system of Micro Aerial Vehicle (MAV) in an outdoor environment, which has the advantages of low-cost and high-precision [1]. By combining GPS with the inertial measurement unit (IMU), the GPS/IMU localization system can obtain the meter-level accuracy [2]. However, GPS cannot be used in the indoor environment [3] because of the signal shading, obstacles and environmental disturbances. Moreover, the localization precision and reliability for the indoor localization system are much more essential. Generally, the indoor localization system can be divided into non-cooperative localization technology and cooperative localization technology based on whether alternative sensors are introduced to provide additional information.

In the non-cooperative localization type, simultaneous localization and mapping (SLAM) is the most important technology, which includes lidar SLAM(L-SLAM) and visual SLAM (V-SLAM). The L-SLAM technology is very mature on unmanned ground vehicle (UGV), which has the advantages of high speed and reliability since the lidar can measure the precise distance and angle of the obstacles. Furthermore, the filters such as extended Kalman filter (EKF) [4] or particle filter (PF) [5] are used to obtain the accurate localization. L-SLAM has been applied in MAV system in recent years gradually. In literature [6], the reliable L-SLAM is realized on MAV system based on miniature 3D-lidar, and the proposed system can locate the MAV in unknown environments without GPS. Some similar systems are designed based on low-cost 2D-lidar since it is much cheaper and lighter. However, its low refresh frequency results in the matching error during the attitude change and high speed flight. V-SLAM is based on mono camera, RGB-D camera and stereo camera, which uses the graph optimization algorithm to minimize the projection error of key-points. Several V-SLAM systems have been proposed in recent years such as Semi-direct visual odometry (SVO) [7] and oriented FAST and rotated brief SLAM (ORB-SLAM) [8]. These two strategies have been applied in many MAV systems, which can realize precise localization based on the high-performance image process unit. However, the robustness issue is an inevitable problem of these pure vision-based methods, due to the fact that image tracking easily fails in the environments with poor visual features. Therefore, research through fusing V-SLAM with other sensors to achieve robust robot navigation is highly demanded.

In the cooperative localization type, base station, motion capture system and 2D markers are utilized as additional sensors. The requirement for on-board sensor and processor is much lower than V-SLAM which makes it suitable for the localization in a particular area. Traditional base station cooperative localization technology uses several signal stations to locate the beacon attached on the target via wireless fidelity (WiFi), ZigBee or radio frequency identification which is very susceptible to the signal multi-path interference, and can only provide meter localization accuracy [9]. Ultra wideband (UWB) is a short-range radio technology, which has been used for indoor localization in recent years. In contrast to ZigBee or WiFi, the localization via UWB is done with the time of flight methodology so it has good anti-interference capability and centimetre localization accuracy. In reference [10], the location information of UWB is introduced in the GPS/IMU navigation system which enhances the reliability of the integrated navigation in GPS inapplicable environment. However, UWB is still affected by the metal shielding and absorption. Meanwhile, the cost of it is much higher than other base station cooperative localization technology. Motion capture cooperative localization technology has high reliability and localization accuracy, which is widely used by movie-making, computer vision and control. For example, The Vicon MX motion capture system is a popular high end commercial solution for the development of autonomous aerial robotic [11]. However, the high cost of the motion capture system limits it only in adaptive industrial or laboratory environments; 2D markers cooperative localization technology is typically introduced in an environment where localization and navigation are needed for robots using low cost machine vision. It can provide accurate six-degrees-of-freedom information of the camera. Meanwhile, it is more reliable and faster than V-SLAM since the markers can be easily identified from a wider range of viewpoints. Therefore, 2D marker array can be used in featureless indoor environments (such as laboratory or corridors) to build a large-scale and cost-effective localization system. For example, the Sky-Trax system uses hundreds of 2D markers to locate the position of materials and equipments inside warehouses and factory [12].

In Sky-Trax, the 2D marker array is regularly arranged so it is easy to artificially calibrate the position of each marker, but in some cases the markers are irregular, which makes accurate calibration very difficult. Therefore, the automatic mapping of the large-scale 2D marker array is very important in practical application. A straightforward way is to implement the SLAM framework as in the literature [13]. In literature [14,15], the authors use odometer/IMU to estimate the robot’s trajectory. Once the camera trajectory is accurately obtained, marker locations are obtained by triangulation. In literature [16] a graph is used to describe the geometric relationship of each marker, which is updated dynamically. Whenever a pair of markers are seen in a frame, their relative position is updated and if it is better than the previous one, it is replaced. For localization, they only consider for localization one marker from all visible ones. However, using all visible markers at the same time can lead to a better localization result. Literature [17] designed a simple marker-based localization system, and the markers are added into the map gradually through the Tf package in robot operate system (ROS) [18]. Although the system can realize real-time online map building, it has a serious problem of mapping distortion due to lack of correction. Literature [19] presents a monocular visual-inertial EKF-SLAM system based on 2D markers. Since only 2D markers and IMU are used, drift may occur if the markers are lost for a long time. Moreover, it requires increasing computational resources to handle covariance matrices that are expanded as more markers are added. Literature [20] proposes a localization method considering all visible markers and handling the mapping as a variant of the sparse bundle adjustment problem. The proposed system can generate an accurate map, but the algorithm runs off-line and requires a large amount of computation.

In this paper, a multi-sensor fusion indoor localization system (MMFL) based on ArUco marker is proposed. The proposed ArUco Mapping algorithm can build and correct the map online with Grubbs criterion and K-mean clustering algorithm, which avoids the map distortion due to lack of correction. In order to realize the localization in marker undetectable situation, the federated Kalman filter with adaptive information coefficient is utilized to synthesize multi-source information from marker, optical flow, ultrasonic and the inertial sensor. Thus, the MMFL system can effectively reduce the position drift due to the long-term loss of markers. The presented system not only gives satisfying localization performance, but also has the potential to expand other localization sensors (such as visual odometry, UWB, and lidar) to further improve the localization performance. The on-board MMFL system can be easily realized on a low cost and low power consumption hardware platform including a Raspberry Pi Zero and two STM32 micro controllers produced by STMicroelectronics. The experimental results show that the speed estimation result is better than that of the Px4flow sensor [21], and the proposed system has the centimeter level errors in positioning and mapping to the manual calibration map.

The rest of this paper is organized as follows. Section 2 introduces the core modules in the proposed MMFL system, including the mapping module and speed estimation module described in Section 2.1, the attitude estimation module described in Section 2.2, and the multi-sensor fusion module described in Section 2.3. Section 3 introduces the hardware design and experimental results of the MMFL system. The experiment results of the proposed mapping algorithm is stated in Section 3.1, the experiment result of the multi-sensor fusion algorithm is stated in Section 3.2, the comparison experiment with ORB-SLAM2 is stated in Section 3.3 and the experiments of comparing the hovering performance with Px4flow sensor is stated in Section 3.4.

## 2. Multi-Sensor Fusion Indoor Localization System Based on ArUco Marker

**Figure 1:**
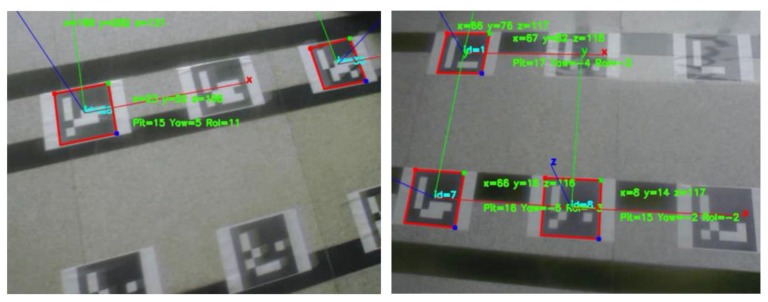
Abnormal Detection Caused by Camera Shake and Reflection.

Generally, the ArUco marker can provide precise localization information with less CPU consumption. However, the tilting and shaking of MAV may cause localization errors, discontinuous positioning, even miss detecting since the camera is mounted on it. Figure 1 shows the cases of abnormal detection caused by shaking and reflecting light, in which most markers can not be detected in these situations and the vertices of the identified markers also have a position error. Therefore, it is straightforward to consider introducing additional sensors to get a satisfying localization result. Thus, a multi-sensor fusion structure combining markers, IMU, ultrasonic and optical flow by the federated Kalman filter is proposed in order to decrease the effect of strong illumination and low quality images.

**Figure 2:**
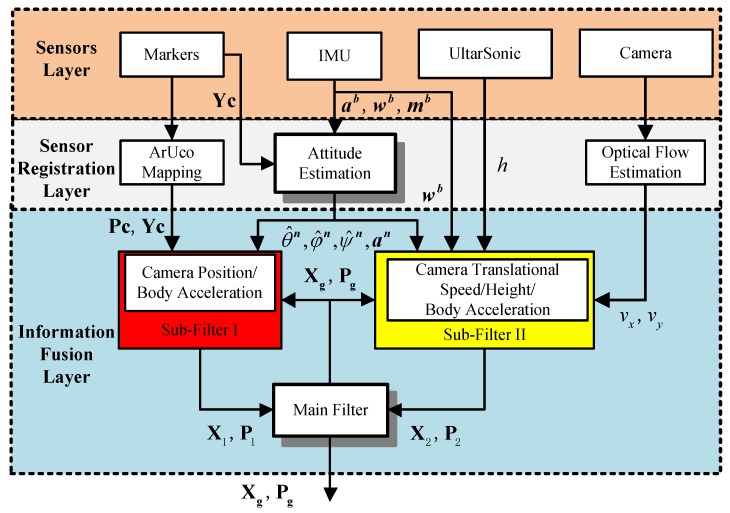
Flow Chart of Multi-Heterogeneous Sensors Layer.

The flow chart of the MMFL system proposed in this paper is shown in Figure 2. The sensor layer is composed of a variety of heterogenous sensors including markers, IMU, ultrasonic and camera. It is possible to further improve the performance by adding other sensors (such as UWB beacon, barometer or lidar). In the sensor registration layer, the proposed ArUco Mapping module uses an online mapping algorithm to realize the camera localization, the attitude estimation of MAV is based on the EKF [22], and the pyramid Lucas-Kanade (PLK) optical flow algorithm is utilized to estimate the translational speed of camera. The information fusion layer is realized by a federated Kalman filter (FK) [23], which contains two sub-filters (SF) and one main filter (MF). The camera position and body acceleration are input SF-I to estimate the position and speed of MAV. The translational speed estimated by optical flow, the height measured by ultrasonic and the body acceleration are input SF-II to estimate the same statement. Finally, the MF synthesizes two SFs’ results for an optimal estimation.

### 2.1. Visual Algorithms in Sensor Registration Layer

**ArUco Mapping:** containing 1024 different modified Hamming codes, the ArUco marker [24] is capable of providing detection, recognition and six-degree-of-freedom (6Dof) information of camera, which is used to give the precise position of MAV in this paper. During location of a large area, a precise map containing the poses of markers is required. The direct way to build this map is to manually calibrate each marker. Obviously, manual mapping is extremely difficult and impossible for an irregular marker array, which is time-consuming and error-prone when calibrating hundreds of markers. A more appropriate way is to automatically build the map with the visual information. Based on this idea, the proposed ArUco Mapping algorithm is described as below.

Define the marker coordinate as *m*, whose origin locates at the center of marker, the *y*-axis points in the forward direction, the *z*-axis goes through the center and the *x*-axis points to the right direction. Define the map coordinate as *n*, which is coincided with the coordinate of the first detected marker. The state vectors of Marker-*i* in coordinate *n* include the center position $p_{i}^{n}=\left(mx_{i}^{n},my_{i}^{n},mz_{i}^{n}\right)$ and orientation $\gamma_{i}^{n}$ in radian. For the markers are set on the flat floor in this paper so the $mz_{i}^{n}$ of all markers are assumed to be zero. In addition, the camera’s position and orientation calculated by Marker-*i* in coordinate *m* is $p_{i}^{m}=\left(x_{i}^{m},y_{i}^{m},z_{i}^{m}\right)$ and $\gamma_{i}^{m}$, the camera’s position and orientation calculated by Marker-*i* in coordinate *n* is $P_{i}^{n}=\left(x_{i}^{n},y_{i}^{n},z_{i}^{n}\right)$, $Y_{i}^{n}$.

(1) $$Y_{i}^{n}=\gamma_{i}^{n}+\gamma_{i}^{m}$$

(2) $$P_{i}^{n}=p_{i}^{n}+R\left(\gamma_{i}^{n}\right)p_{i}^{m}$$

with $R\left(\gamma \right)=\left[\begin{array}{ccc} \cos(\gamma ) & \sin(\gamma ) & 0\\ -\sin(\gamma ) & \cos(\gamma ) & 0\\ 0 & 0 & 1 \end{array}\right].$

It is assumed that the position and orientation of the MAV in map coordinate calculated by each marker are assumed to be same, thus the $P^{n}$ and $Y^{n}$ calculated by two markers should be same in this case. The newly detected Marker-*j*’s position $p_{j}^{n}$ and orientation $\gamma_{j}^{n}$ in coordinate *n* can be calculated by Equations (3)–(6) based on the position and orientation of Marker-*i*.

(3) $$Y_{i,j}^{n}=\gamma_{i}^{n}+\gamma_{i}^{m}=\gamma_{j}^{n}+\gamma_{j}^{m}$$

(4) $$\gamma_{j}^{n}=\gamma_{i}^{n}+\gamma_{i}^{m}-\gamma_{j}^{m}$$

(5) $$P_{i,j}^{n}=p_{i}^{n}+R\left(\gamma_{i}^{n}\right)p_{i}^{m}=p_{j}^{n}+R\left(\gamma_{j}^{n}\right)p_{j}^{m}$$

(6) $$p_{j}^{n}=p_{i}^{n}+R\left(\gamma_{i}^{n}\right)p_{i}^{m}-R\left(\gamma_{j}^{n}\right)p_{j}^{m}$$

The presented mapping algorithm uses the first detected marker to initialize the map *M* then the newly detected marker is added into the map gradually. In addition, the position and orientation of each marker is calculated and restored in its own point cloud ζ. The Grubbs criterion [25] is used to remove the outliers from point cloud when its size is bigger than *S*, then the *K*-mean cluster [26] is adopted to find the center of *K* clusters in the point cloud. The most likely center P*, Y* synthesize each clusters’ center with the corresponding weight, which is determined by the degree of data concentration. P*, Y* are used to correct the markers’ position Pc and orientation Yc in map *M* based on step β. After each map correction, a point will be removed from its point cloud if the pose error is bigger than *R* and a marker will be “fixed” if the pose error between two revisions is smaller than *F*. Finally, the ArUco SDK is used to calculate the camera’s position Pc and orientation Yc based on the map. The pseudo-code of ArUco Mapping (AM) is given in the following Algorithm 1.

**Pyramid Lucas-Kanade Optical Flow:** optical flow is the velocity vector caused by camera movement, which is used to estimate the speed of MAV in this paper. The traditional method for measuring optical flow is the Lucas-Kanade method [27], which assumes that the image displacement between two adjacent frames is small. Therefore, the disadvantage of this method is that the matched pixel candidates cannot be found in a small neighborhood during fast motion. A common improvement of the LK method is to calculate the optical flow with pyramids, which is known as the PLK method [28]. The PLK method compresses the size of the image on different pyramid levels, so that the optical flow caused by fast motion can be calculated on the top level. For this reason, the PLK method is able to estimate the optical flow during fast motion. The PLK method initializes the optical flow on the highest level. Set $d^{L}$ is the optical flow calculated by the LK method at level *L* and $g^{L}$ is the predicted optical flow based on the upper level. Thus the updated optical flow of level *L* is $k^{L}=g^{L}+d^{L}$, and the predicted optical flow of level *L* + 1 is $g^{L+1}=2\times k^{L}$. Through repeating this method to the lowest level of the pyramids, the final optical flow can be calculated.

**Algorithm 1** Pseudo-Code of the ArUco Mapping Algorithm**Input:**M: current map, ζ: point cloud, Π: set of detected marks, *S*: size of point cloud, β: correction step, *L*: number of markers in map, *N*: number of detected markers, *K*: number of cluster, *R*: culling threshold of point cloud, *F*: marker fixed threshold **Output:**Pm: markers’ position in map, Ym: markers’ orientation in map, Pc: camera’s position, Yc: camera’s orientation;
1:***Map initialize:***    initialize the map coordinate by the firstly detected marker2:**if**N>0**then**3: **for** i=0 to *N* in Π **do**4:  **if** Π(i) not in M **then**5:   **for** j=1 to *N* **do**6:    **if** Π(j) in M and i!=j **then**7:     ***Add the newly detected Marker in to map:***    Calculate $P_{j}^{n}$ and $\gamma_{j}^{n}$ by Equations (3)–(6) and initialize ζ(i);8:    **end if**9:   **end for**10:  **else**11:   **for** j=1 to *N* **do**12:    **if** Π(j) in M and i!=j **then**13:     ***Point cloud sampling:***    Calculate $P_{j}^{n}$ and $\gamma_{j}^{n}$ by Equations (3)–(6) and add it into ζ(i);14:    **end if**15:   **end for**16: 17:   ***Remove outliers:***    $\zeta^{*}\left(i\right)$ = Grubbs(ζ(i));18: 19:   ***Find the center of the point cloud:***20:   The center of each cluster:    $C_{n}^{p},C_{n}^{Y}\left(n=1,2,...,K\right)=$ K-means$\left(\zeta^{*}\left(i\right),K\right)$;21:   **for** n=1 to *K* **do**22:    Calculate the lumpiness $\delta_{n}^{p},\delta_{n}^{Y}$ of $C_{n}^{p},C_{n}^{Y}$, based on the average distance between all points and the centers;23:   **end for**24:   Find the maximum value $\ell^{p},\ell^{Y}$ of lumpiness $\delta_{n}^{p},\delta_{n}^{Y}\left(n=1,2,...,K\right)$;25:   Calculate the final center and orientation of the point cloud P*(i)=∑n=1Kℓpℓpδnpδnp∑m=1KℓpℓpδmpδmpCnp,Y*(i)=∑n=1KℓYℓYδnYδnY∑m=1KℓYℓYδmYδmYYCnY;26: 27:   ***Map correction:***    Use $P^{*}\left(i\right),Y^{*}\left(i\right)$ to modify Pm(i),Ym(i) with step β;28: 29:   ***Point cloud clipping:***30:   **for** k=1 to all points in ζ(i) **do**31:    **if** ${∥\zeta \left(i\right)}_{k}{-\mathrm{Pm}\left(i\right)∥}_{L2}>R$ **then**32:     Remove this point from ζ(i);33:    **end if**34:   **end for**35: 36:   ***Fix marker:***37:   **if**$∥P^{*}{\left(i\right)-\mathrm{Pm}\left(i\right)∥}_{L2}<F$ **then**38:    Fix this marker in map and do not correct it later;39:   **end if**40:  **end if**41: **end for**42: 43: ***Calculate Camera position:***44: **for** i=1 to all Π in M **do**45:  Calculate $P_{i}^{n}$ and $Y_{i}^{n}$ by Equations (1) and (2);46: **end for**47: calculate the average value of $P_{i}^{n}\left(i=1,2,\ldots ,N\right)$ as $\mathrm{Pc}=(P_{x}^{c},P_{y}^{c},P_{z}^{c})$, calculate the average value of $Y_{i}^{n}\left(i=1,2,\ldots ,N\right)$ as Yc;48:**end if**


Since the camera faces the ground, the rotation of pitch, roll will generate the rotating optical flow, which can be compensated with IMU to reflect the real translational speed. Define the body coordinate as *b*, which is a non-inertial coordinate system associated with the vehicle’s center of gravity. The *x*-axis points in the forward direction, the *z*-axis down through the vehicle and the *y*-axis completes the right-hand coordinate system. Set $d_{x}$, $d_{y}$ as the average of the final optical flow calculated by the PLK method, the compensated optical flow Dx, Dy can be calculated as below.

(7) $$D_{x}=d_{x}-w_{x}^{b}W_{x}/\mu$$

(8) $$D_{y}=d_{y}-w_{y}^{b}W_{y}/\mu$$

where $w_{x}^{b}$ and $w_{y}^{b}$ are the angular speeds measured by the gyroscope. $W_{x}$ is the number of image rows, $W_{y}$ is the number of image columns, μ is the field of view and the translational speed of camera is given as below:

(9) $$v_{x}=2hD_{x}/W_{x}\tan\left(\mu /2\right)$$

(10) $$v_{y}=2hD_{y}/W_{y}\tan\left(\mu /2\right)$$

where *h* is the distance to the ground level given by the ultrasonic sensor.

### 2.2. Attitude Estimation Module in Sensor Registration Layer

**Figure 3:**
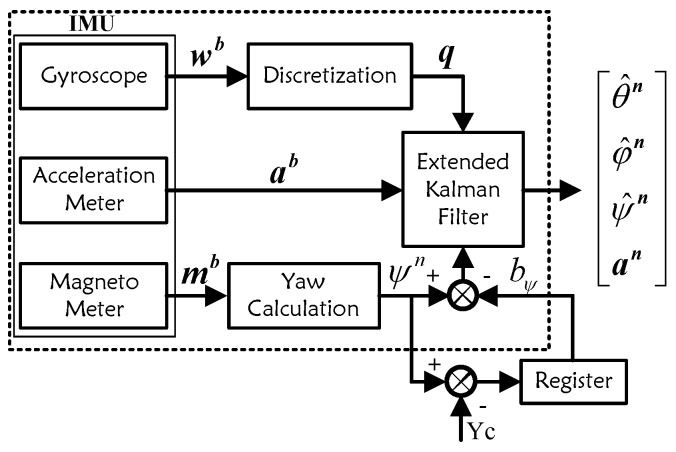
Flow Chart of Attitude Estimation Module.

Figure 3 shows the flow chart of the attitude estimation module in sensor registration layer, and an IMU includes 3-axis gyroscope, 3-axis acceleration meter and 3-axis magneto meter is used here. $w^{b}\left(k\right)=\left[w_{x}^{b}\left(k\right),w_{y}^{b}\left(k\right),w_{z}^{b}\left(k\right)\right]$ is the angular speed measured by gyroscope, $a^{b}\left(k\right)=\left[a_{x}^{b}\left(k\right),a_{y}^{b}\left(k\right),a_{z}^{b}\left(k\right)\right]$ is the body acceleration measured by acceleration meter, $m^{b}\left(k\right)=\left[m_{x}^{b}\left(k\right),m_{y}^{b}\left(k\right),m_{z}^{b}\left(k\right)\right]$ is magnetic field measured by magnetometer, $\xi^{n}\left(k\right)={\left[{\hat{\phi }}^{n}\left(k\right),{\hat{\theta }}^{n}\left(k\right),{\hat{\psi }}^{n}\left(k\right),a^{n}\left(k\right)\right]}^{T}$ is the estimated attitude and acceleration in map coordinate *n*.

**State Model:** the attitude estimation is based on the quaternion [29] and EKF. The state vector $x\left(k\right)={\left[q\left(k\right),b\left(k\right)\right]}^{T}={\left[q_{0}\left(k\right),q_{1}\left(k\right),q_{2}\left(k\right),q_{3}\left(k\right),b_{x}\left(k\right),b_{y}\left(k\right),b_{z}\left(k\right)\right]}^{T}$ consists of two parts: quaternion and gyro bias, and the system function is given below:

(11) $$x\left(k\right)=f\left(x\left(k-1\right),k-1\right)=\left[\begin{array}{c} (I+\left(T/2\right)\Omega_{w}\left(k-1\right))q\left(k-1\right)\\ b(k-1) \end{array}\right]+\sigma \left(k-1\right)$$

where $\Omega_{w}\left(k-1\right)=\left[\begin{array}{cccc} 0 & -w_{x}^{b}\left(k\right)+b_{x}\left(k-1\right) & -w_{y}^{b}\left(k\right)+b_{y}\left(k-1\right) & -w_{z}^{b}\left(k\right)+b_{z}\left(k-1\right)\\ w_{x}^{b}\left(k\right)-b_{x}\left(k-1\right) & 0 & w_{z}^{b}\left(k\right)-b_{z}\left(k-1\right) & -w_{y}^{b}\left(k\right)+b_{y}\left(k-1\right)\\ w_{y}^{b}\left(k\right)-b_{y}\left(k-1\right) & -w_{z}^{b}\left(k\right)+b_{z}\left(k-1\right) & 0 & w_{x}^{b}\left(k\right)-b_{x}\left(k-1\right)\\ w_{z}^{b}\left(k\right)-b_{z}\left(k-1\right) & w_{y}^{b}\left(k\right)-b_{y}\left(k-1\right) & -w_{x}^{b}\left(k\right)+b_{x}\left(k-1\right) & 0 \end{array}\right]$, *T* is the sample period, I is the identity matrix of appropriate dimension, σ(k) is gaussian white noise of system and $E[\sigma \left(k-1\right)\sigma {\left(k-1\right)}^{T}]=Q\left(k-1\right)$. 

**Measurement Model:** the measurement $z\left(k\right)={\left[a_{x}^{b}\left(k\right),a_{y}^{b}\left(k\right),a_{z}^{b}\left(k\right),\psi^{n}\left(k\right)-b_{\psi }\left(k\right)\right]}^{T}$ includes the body acceleration and yaw in map coordinate, $\psi^{n}\left(k\right)$ is the yaw in earth coordinate and it can be calculated by the measurement of magneto meter:

(12) $$\psi^{n}\left(k\right)=\arctan\left(m_{y}^{n}\left(k\right)/m_{x}^{n}\left(k\right)\right)$$

(13) $$m_{x}^{n}\left(k\right)=m_{x}^{b}\left(k\right)\cos\hat{\theta }\left(k\right)+m_{y}^{b}\left(k\right)\sin\hat{\phi }\left(k\right)\sin\hat{\theta }\left(k\right)-m_{z}^{b}\cos\hat{\phi }\left(k\right)\sin\hat{\theta }\left(k\right)$$

(14) $$m_{y}^{n}\left(k\right)=m_{y}^{b}\left(k\right)\cos\hat{\phi }\left(k\right)+m_{z}^{b}\left(k\right)\sin\hat{\phi }\left(k\right)$$

In addition, there is a bias between map coordinate and earth coordinate since the map coordinate is initialized by the first detected marker. Define this bias as $b_{\psi }\left(k\right)$ and it can be calculated as below:

$$b_{\psi }\left(k\right)=\left\{\begin{array}{cc} \psi^{n}\left(k\right)-Yc\left(k\right) & \mathrm{If}\;\mathrm{any}\;\mathrm{markers}\;\mathrm{are}\;\mathrm{detected}\;\mathrm{in}\;\mathrm{the}\;\mathrm{image}\\ b_{\psi }\left(k-1\right) & \mathrm{Otherwise} \end{array}\right.$$

Therefore, the measurement function is given below:

(15) $$z\left(k\right)=h\left(x\left(k\right),k\right)+v\left(k\right)=\left[\begin{array}{c} -2g(q_{1}\left(k\right)q_{3}\left(k\right)-q_{0}\left(k\right)q_{2}\left(k\right))\\ -2g(q_{2}\left(k\right)q_{3}\left(k\right)+q_{0}\left(k\right)q_{1}\left(k\right))\\ -g({q_{0}\left(k\right)}^{2}-{q_{1}\left(k\right)}^{2}-{q_{2}\left(k\right)}^{2}+{q_{3}\left(k\right)}^{2})\\ \arctan(-\frac{2(q_{1}\left(k\right)q_{2}\left(k\right)-q_{0}\left(k\right)q_{3}\left(k\right))}{{q_{0}\left(k\right)}^{2}-{q_{1}\left(k\right)}^{2}+{q_{2}\left(k\right)}^{2}-{q_{3}\left(k\right)}^{2}}) \end{array}\right]+v\left(k\right)$$

where v(k) is the measurement noise and $E[v\left(k\right)v{\left(k\right)}^{T}]=R\left(k\right)$. 

**Attitude Estimation:** the extended Kalman filter equations of the attitude estimation module are given below.

(16) $$\hat{x}\left(k,k-1\right)=f\left(\hat{x}\left(k-1\right),k-1\right)$$

(17) $$P\left(k,k-1\right)=\nabla f_{x(k-1)}P\left(k-1\right)\nabla f_{x(k-1)}^{T}+Q\left(k\right)$$

(18) $$K\left(k\right)=P\left(k,k-1\right)\nabla h_{x(k-1)}^{T}{\left(\nabla h_{x(k-1)}P\left(k,k-1\right)\nabla h_{x(k-1)}^{T}+R\left(k\right)\right)}^{-1}$$

(19) $$\hat{x}\left(k\right)=\hat{x}\left(k,k-1\right)+K\left(k\right)\left[z\left(k\right)-h\left(\hat{x}\left(k,k-1\right),k\right)\right]$$

(20) $$P\left(k\right)=(I-K\left(k\right)\nabla h_{x(k-1)})P\left(k,k-1\right)$$

where P(k) is the covariance matrix and K(k) is the Kalman gain matrix, Q(k) and R(k) are the system and measurement covariance matrices respectively. As the appropriate matrices Q(k) and R(k) cannot be chosen based on classical theories, they are usually tuned experimentally by a trial-and-error method. The Jacobi matrix of the nonlinear system function and measurement function are given below.

(21) $$\nabla f_{x(k)}=\left[\begin{array}{ccccccc} 1 & -T{\hat{w}}_{x}\left(k\right)/2 & -T{\hat{w}}_{y}\left(k\right)/2 & -T{\hat{w}}_{z}\left(k\right)/2 & Tq_{1}\left(k\right)/2 & Tq_{2}(k/2 & Tq_{3}\left(k\right)/2\\ T{\hat{w}}_{x}\left(k\right)/2 & 1 & T{\hat{w}}_{z}\left(k\right)/2 & -T{\hat{w}}_{y}\left(k\right)/2 & -Tq_{0}\left(k\right)/2 & Tq_{3}\left(k\right)/2 & -Tq_{2}\left(k\right)/2\\ T{\hat{w}}_{y}\left(k\right)/2 & -T{\hat{w}}_{z}\left(k\right)/2 & 1 & T{\hat{w}}_{x}\left(k\right)/2 & -Tq_{3}\left(k\right)/2 & -Tq_{0}\left(k\right)/2 & Tq_{1}\left(k\right)/2\\ T{\hat{w}}_{z}\left(k\right)/2 & T{\hat{w}}_{y}\left(k\right)/2 & -T{\hat{w}}_{x}\left(k\right)/2 & 1 & Tq_{2}\left(k\right)/2 & -Tq_{1}\left(k\right)/2 & -Tq_{0}\left(k\right)/2\\ 0 & 0 & 0 & 0 & 1 & 0 & 0\\ 0 & 0 & 0 & 0 & 0 & 1 & 0\\ 0 & 0 & 0 & 0 & 0 & 0 & 1 \end{array}\right]$$

(22) $$\nabla h_{x(k)}=\left[\begin{array}{ccccccc} 2gq_{2}\left(k\right) & -2gq_{3}\left(k\right) & 2gq_{0}\left(k\right) & -2gq_{1}\left(k\right) & 0 & 0 & 0\\ -2gq_{1}\left(k\right) & -2gq_{0}\left(k\right) & -2gq_{3}\left(k\right) & -2gq_{2}\left(k\right) & 0 & 0 & 0\\ -2gq_{0}\left(k\right) & 2gq_{1}\left(k\right) & 2gq_{2}\left(k\right) & 2gq_{3}\left(k\right) & 0 & 0 & 0\\ \frac{2q_{3}\left(k\right)S_{1}+4q_{0}\left(k\right)S_{2}}{{S_{1}}^{2}+4{S_{2}}^{2}} & \frac{-2q_{2}\left(k\right)S_{1}-4q_{1}\left(k\right)S_{2}}{{S_{1}}^{2}+4{S_{2}}^{2}} & \frac{-2q_{1}\left(k\right)S_{1}+4q_{2}\left(k\right)S_{2}}{{S_{1}}^{2}+4{S_{2}}^{2}} & \frac{2q_{0}\left(k\right)S_{1}-4q_{3}\left(k\right)S_{2}}{{S_{1}}^{2}+4{S_{2}}^{2}} & 0 & 0 & 0 \end{array}\right]$$

where $\hat{w}\left(k\right)=\left[\begin{array}{c} {\hat{w}}_{x}\left(k\right)\\ {\hat{w}}_{y}\left(k\right)\\ {\hat{w}}_{z}\left(k\right) \end{array}\right]=\left[\begin{array}{c} w_{x}^{b}\left(k\right)-b_{x}\left(k\right)\\ w_{y}^{b}\left(k\right)-b_{y}\left(k\right)\\ w_{z}^{b}\left(k\right)-b_{z}\left(k\right) \end{array}\right]$ is the angular velocity after compensated the gyroscope-drift, $S_{1}={q_{0}\left(k\right)}^{2}-{q_{1}\left(k\right)}^{2}+{q_{2}\left(k\right)}^{2}-{q_{3}\left(k\right)}^{2}$, $S_{2}=q_{1}\left(k\right)q_{2}\left(k\right)-q_{0}\left(k\right)q_{3}\left(k\right)$ and *g* is the gravity.

**Normalization:** the quaternion needs to be normalized at the end of each filter cycle. So the final output of the filter is given as below:

(23) $$q_{i}\left(k\right)=q_{i}\left(k\right)/\sqrt{q_{0}{\left(k\right)}^{2}+q_{1}{\left(k\right)}^{2}+q_{2}{\left(k\right)}^{2}+q_{3}{\left(k\right)}^{2}},\left(i=0,1,2,3\right)$$

(24) $$\xi^{n}\left(k\right)=\left[\begin{array}{c} {\hat{\theta }}^{n}\left(k\right)\\ {\hat{\varphi }}^{n}\left(k\right)\\ {\hat{\psi }}^{n}\left(k\right)\\ a^{n}\left(k\right) \end{array}\right]=\left[\begin{array}{c} \arcsin(2\left(q_{2}\left(k\right)q_{3}\left(k\right)+q_{0}\left(k\right)q_{1}\left(k\right)\right))\\ \arctan(\frac{2(q_{1}\left(k\right)q_{3}\left(k\right)-q_{0}\left(k\right)q_{2}\left(k\right))}{{q_{0}\left(k\right)}^{2}-{q_{1}\left(k\right)}^{2}-{q_{2}\left(k\right)}^{2}+{q_{3}\left(k\right)}^{2}})\\ \arctan(-\frac{2(q_{1}\left(k\right))q_{2}\left(k\right)-q_{0}\left(k\right)q_{3}\left(k\right))}{{q_{0}\left(k\right)}^{2}-{q_{1}\left(k\right)}^{2}+{q_{2}\left(k\right)}^{2}-{q_{3}\left(k\right)}^{2}})\\ C_{b}^{n}a^{b}\left(k\right) \end{array}\right]$$

where

(25) $$C_{b}^{n}=\left[\begin{array}{ccc} {q_{0}\left(k\right)}^{2}+{q_{1}\left(k\right)}^{2}-{q_{2}\left(k\right)}^{2}-{q_{3}\left(k\right)}^{2} & 2(q_{1}\left(k\right)q_{2}\left(k\right)-q_{0}\left(k\right)q_{3}\left(k\right)) & 2(q_{1}\left(k\right)q_{3}\left(k\right)+q_{0}\left(k\right)q_{2}\left(k\right))\\ 2(q_{1}\left(k\right)q_{2}\left(k\right)+q_{0}\left(k\right)q_{3}\left(k\right)) & {q_{0}\left(k\right)}^{2}-{q_{1}\left(k\right)}^{2}+{q_{2}\left(k\right)}^{2}-{q_{3}\left(k\right)}^{2} & 2(q_{2}\left(k\right)q_{3}\left(k\right)+q_{0}\left(k\right)q_{1}\left(k\right))\\ 2(q_{1}\left(k\right)q_{3}\left(k\right)-q_{0}\left(k\right)q_{2}\left(k\right)) & 2(q_{2}\left(k\right)q_{3}\left(k\right)-q_{0}\left(k\right)q_{1}\left(k\right)) & {q_{0}\left(k\right)}^{2}-{q_{1}\left(k\right)}^{2}-{q_{2}\left(k\right)}^{2}+{q_{3}\left(k\right)}^{2} \end{array}\right]$$

### 2.3. Information Fusion of Multi-Heterogeneous Sensors

The information fusion layer is designed to fuse the information received from the upper layers. A federated filter is used here based on the advantage of fusing heterogeneous sensors data, which allows the sensor layer to measure the same states with different sensors (for example ultrasonic and ArUco Mapping can both give the height of MAV). Additionally, it is easy to expand other sensors to further improve the localization precision and provide a good redundancy. The federated filter is comprised of one main filter (MF) and several sub-filters (SF), which is fault tolerant and flexible. The state and covariance matrix of each sub-filter are sent to the MF and the MF fuses the two estimations to get the optimal result.

**State Model**: A distributed system with controlled input and noise is given as follows:

(26) $$X_{g}\left(k\right)=AX_{g}\left(k-1\right)+\mathrm{BU}\left(k-1\right)+\Gamma W\left(k-1\right)$$

(27) $$A=\left[\begin{array}{ccc} F & 0_{3\times 3} & 0_{3\times 3}\\ 0_{3\times 3} & F & 0_{3\times 3}\\ 0_{3\times 3} & 0_{3\times 3} & F \end{array}\right]$$

(28) $$B=\left[\begin{array}{ccc} G & 0_{3\times 1} & 0_{3\times 1}\\ 0_{3\times 1} & G & 0_{3\times 1}\\ 0_{3\times 1} & 0_{3\times 1} & G \end{array}\right]$$

(29) $$U\left(k-1\right)=a^{n}\left(k-1\right)$$

with F=1T−T2−T2220T−T001, G=T2T222T0, where *T* is the sample period, A is the state-transition matrix, B is the controlled input matrix, $0_{3\times 3}$ is the zero matrix with the appropriate dimension, U(k−1) is the controlled input, Γ is the noise drive matrix, W(k−1) is the gaussian white noise and $E[W\left(k-1\right)W{\left(k-1\right)}^{T}]=Q\left(k-1\right)$. The state vector $X_{g}\left(k\right)={\left[x\left(k\right),\dot{x}\left(k\right),\tau_{x}\left(k\right),y\left(k\right),\dot{y}\left(k\right),\tau_{y}\left(k\right),z\left(k\right),\dot{z}\left(k\right),\tau_{z}\left(k\right)\right]}^{T}$ includes position, speed, and acceleration bias in the map coordinate.

**Measurement Model**: The measurement of SF-I is the camera position $\mathrm{Pc}\left(k\right)=(P_{x}^{c}\left(k\right),P_{y}^{c}\left(k\right),P_{z}^{c}\left(k\right))$, and the measurement function is:

(30) $$z_{1}\left(k\right)={\left[\begin{array}{ccccccccc} P_{x}^{c}\left(k\right) & 0 & 0 & P_{y}^{c}\left(k\right) & 0 & 0 & P_{z}^{c}\left(k\right) & 0 & 0 \end{array}\right]}^{T}=H_{1}X_{1}\left(k\right)+n_{1}\left(k\right)$$

where $H_{1}=\mathrm{diag}\left(1,0,0,1,0,0,1,0,0\right)$, $X_{1}\left(k\right)$ is the state vector of SF-I, $n_{1}\left(k\right)$ is the measurement noise for marker, and its covariance matrix is $R_{1}\left(k\right)$.

The measurement of SF-II contains the speed estimated by PLK and the height given by ultrasonic. The measurement function is:

(31) $$\begin{array}{c} \begin{array}{c} z_{2}\left(k\right)={\left[\begin{array}{ccccccccc} 0 & V_{x}\left(k\right) & 0 & 0 & V_{y}\left(k\right) & 0 & \frac{h(k)}{\cos{\hat{\varphi }}^{n}\left(k\right)\cos{\hat{\theta }}^{n}\left(k\right)} & 0 & 0 \end{array}\right]}^{T}=H_{2}X_{2}\left(k\right)+n_{2}\left(k\right) \end{array} \end{array}$$

where $\left[\begin{array}{c} V_{x}\left(k\right)\\ V_{y}\left(k\right) \end{array}\right]=\left[\begin{array}{cc} \cos{\hat{\psi }}^{n}\left(k\right) & \sin{\hat{\psi }}^{n}\left(k\right)\\ -\sin{\hat{\psi }}^{n}\left(k\right) & \cos{\hat{\psi }}^{n}\left(k\right) \end{array}\right]\left[\begin{array}{c} v_{x}\left(k\right)\\ v_{y}\left(k\right) \end{array}\right]$, $H_{2}=\mathrm{diag}\left(0,1,0,0,1,0,1,0,0\right)$, $X_{2}\left(k\right)$ is the state vector of SF-II, $n_{2}\left(k\right)$ is the measure noise vector for PLK and ultrasonic, and its covariance matrix is $R_{2}\left(k\right)$.

**Information Fusion:** the standard Kalman filter algorithm is used in the presented MMFL system since the state and measurement functions are both linear, and the federated Kalman filter algorithm [23] is given as below:

(1) The MF initializes ${\hat{X}}_{g}\left(k-1\right),P_{g}\left(k-1\right)$ and $Q_{g}\left(k-1\right)$, then uses the positive coefficient $\beta_{i}\left(k-1\right)$ to distribute the information as follows:
  (32) $${\hat{X}}_{i}\left(k-1\right)={\hat{X}}_{g}\left(k-1\right)$$
  (33) $$P_{i}\left(k-1\right)=\beta_{i}^{-1}\left(k-1\right)P_{g}\left(k-1\right)$$
  (34) $$Q_{i}\left(k-1\right)=\beta_{i}^{-1}\left(k-1\right)Q_{g}\left(k-1\right)$$
  where ${\hat{X}}_{i}\left(k\right)\left(i=1,2\right)$ represents the estimated state vector of each sub-filter, $P_{i}\left(k-1\right)$ and $Q_{i}\left(k-1\right)\left(i=1,2\right)$ represent the corresponding estimated state covariance and process noise covariance, respectively. The coefficient is subject to $\beta_{i}\left(k-1\right)>0$ and $\sum_{i}\beta_{i}\left(k-1\right)=1$.
  According to Equations (32)–(34) the federated filter allocates the global estimation to each SF based on β(k−1), and a smaller information coefficient means less use of the global estimation. For example, the global estimation is average allocated between each SF when $\beta_{i}\left(k-1\right)=0.5\left(i=1,2\right)$ [30]. To make the filter more adaptive, the presented algorithm adopts the variance matrix’s Frobeniuse norm of each SF as the distribution coefficient since the fixed information coefficients can not reflect the accuracy change of SFs. Therefore, a better accurate SF can allocate a larger information coefficient and have a great contribution to the global estimation [31].
  $$\beta_{i}\left(k\right)=\frac{(∥P_{i}\left(k-1\right){{∥}_{F})}^{-1}}{(∥P_{1}{\left(k-1\right)∥}_{F}{)}^{-1}+(∥P_{2}\left(k-1\right){{∥}_{F})}^{-1}}$$
  where ${∥\chi ∥}_{F}=\sqrt{\sum diag(\chi^{T}\chi )}$.
(2) The state prediction of SF-I and SF-II is implemented independently.
  (35) $${\hat{X}}_{i}\left(k,k-1\right)=A{\hat{X}}_{i}\left(k-1\right)+\mathrm{BU}\left(k-1\right)$$
  (36) $${\hat{P}}_{i}\left(k,k-1\right)={\mathrm{AP}}_{i}\left(k-1\right)A^{T}+Q_{i}\left(k-1\right)$$
(3) SF-I and SF-II use the measurement to update the state estimation, respectively.
  (37) $$K_{i}\left(k\right)={\hat{P}}_{i}\left(k,k-1\right)H_{i}^{T}{\left(H_{i}{\hat{P}}_{i}\left(k,k-1\right)H_{i}^{T}+R_{i}\left(k\right)\right)}^{-1}$$
  (38) $${\hat{X}}_{i}\left(k\right)={\hat{X}}_{i}\left(k,k-1\right)+K_{i}\left(k\right)\left(z_{i}\left(k\right)-H_{i}{\hat{X}}_{i}\left(k,k-1\right)\right)$$
  (39) $$P_{i}\left(k\right)=\left(I-K_{i}\left(k\right)H_{i}\right){\hat{P}}_{i}\left(k,k-1\right)$$
(4) The MF fuses the estimations of the two SFs to obtain the optimal result.
  (40) $$P_{g}\left(k\right)={\left(P_{1}^{-1}\left(k\right)+P_{2}^{-1}\left(k\right)\right)}^{-1}$$
  (41) $${\hat{X}}_{g}\left(k\right)=P_{g}\left(k\right)\left(P_{1}^{-1}\left(k\right){\hat{X}}_{1}\left(k\right)+P_{2}^{-1}\left(k\right){\hat{X}}_{2}\left(k\right)\right)$$
  (42) $$Q_{g}\left(k\right)={\left(Q_{1}^{-1}\left(k\right)+Q_{2}^{-1}\left(k\right)\right)}^{-1}$$

A special case is that the markers cannot be detected when MAV is taking off, landing or flying at a low height. To make the filter work properly in those situations, the local estimation of SF-I will be blocked to MF. Thus, the global estimation is ${\hat{X}}_{g}\left(k\right)={\hat{X}}_{2}\left(k\right)$, and the measurement prediction of SF-I is ${\hat{z}}_{1}\left(k\right)=H_{1}{\hat{X}}_{g}\left(k\right)$. Besides, the measurement delay to IMU can be handled by the ring-buffer scheme proposed in [32] for each SF.

## 3. Experiment Result and Analysis

**Figure 4:**
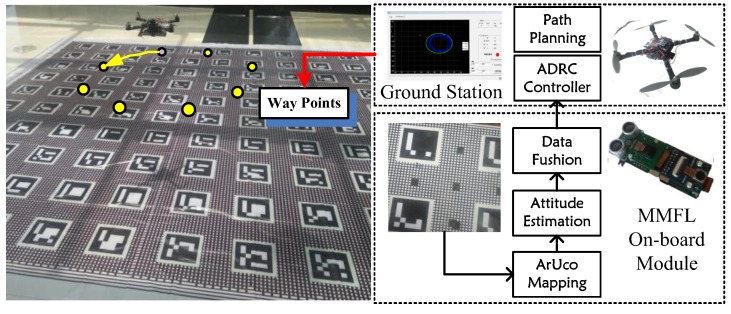
Experiment Site.

In order to verify the proposed MMFL system, a 4 × 4 m experiment site is established in this paper. It contains 100 ArUco markers of 18 × 18 cm in a regular layout with increasing number. The marker array’s center row spacing is 38 cm and the center column spacing is 38.5 cm. The experimental site is shown in Figure 4.

**Figure 5:**
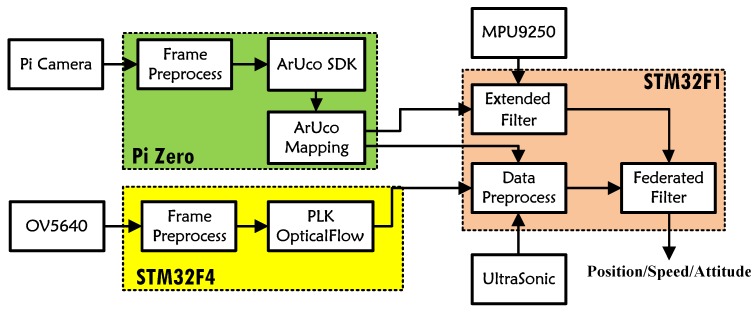
On-board Module Structure Diagram.

In the proposed MMFL system, one Raspberry Pi Zero and two STM32 micro controllers produced by STMicroelectronics are used to build the on-board module, which is smaller than 8×3 cm and lighter than 50 g. The detailed hardware structure is shown in Figure 5. In the proposed on-board module, the Raspberry Pi Zero captures the image from the Pi camera and implements ArUco Mapping at the frequency of 10 Hz. The STM32F4 captures the image from the OV5640 camera and implements speed estimation via PLK at the frequency of 20 Hz. All sensor acquisition (including one MPU9250 inertial sensor and one US-100 ultrasonic) and data fusion are completed in STM32F1 at the frequency of 100 Hz. The STM32F1, STM32F4 and Pi Zero are connected by serial port, and the Mavlink [33] protocol is adopted to guarantee the data transmission. In the software of STM32F1, each sensor has a flag for detecting whether its data is received and the sub-filter uses the measurement to update its state only if its flag is true. The commands and desired way points of MAV are transmitted from the ground station via 2.4 G radio and the real-time 6Dof information of MAV can be observed on the ground station.

### 3.1. Experiment Result of the ArUco Mapping Module

**Figure 6:**
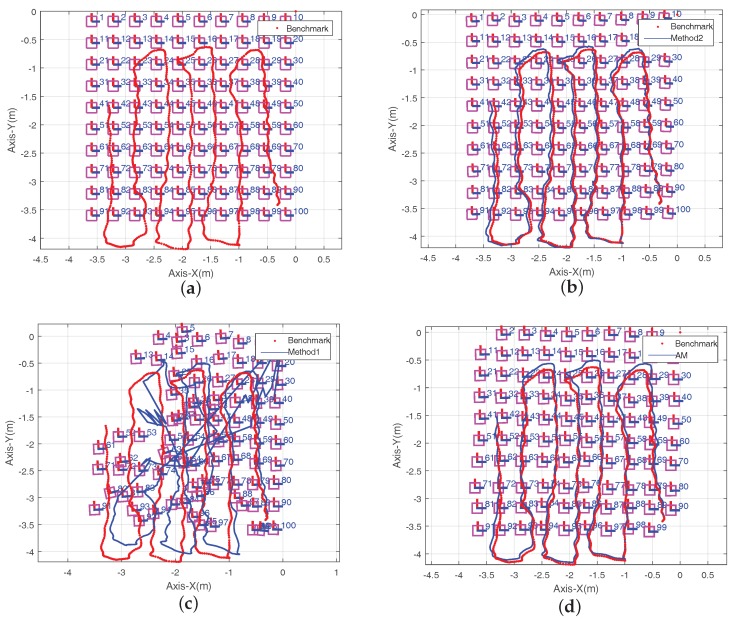
Result of Comparison Experiments. (**a**) Manual Calibrated Map; (**b**) Map of Method 2; (**c**) Map of Method 1; (**d**) Map of the Proposed System.

The experiment in this section is designed to test the performance of the ArUco Mapping (AM). Comparison experiments between the proposed ArUco Mapping in this paper and the mapping systems proposed in reference [18] (noted as Method 1), reference [20] (noted as Method 2) are performed. All the three candidate systems use the same video source as input. Besides, the ArUco SDK is used to calculate the camera position with the different maps generated by the three candidate mapping systems. The trajectory calculated by the manually calibrated map is adopted as the “benchmark” to evaluate the performance of the three candidate systems, since the marker array is regularly arranged and the calibrated error can be omitted. The comparison result is shown in Figure 6.

It can be seen from Figure 6 that the map of the experimental site can be successfully established by Method 2 and the proposed AM system. The mapping result of Method 1 has the catastrophic mistake due to the lack of correction in markers’ pose. Compared with the manual calibrated map, Method 2 has the outstanding mapping performance, however it is running off-line, which takes 10 s to build and optimize the map (executing on IntelRAtom i5-4300U (1.9 GHz) CPU with Ubuntu operating system). Furthermore, two performance indexes are introduced to evaluate the performance of the three candidate mapping systems: estimate accuracy of the marker poses, and estimate accuracy of the camera position. The first one can be evaluated by calculating the Absolute Corner Error (ACE), which computes the mean error between the estimated marker corner locations and the manual calibrated ones. The estimate accuracy of the camera position can be obtained by calculating the Absolute Trajectory Error (ATE), which computes the mean error between the estimated camera position and the benchmark one. The ACE and ATE comparison results of the three candidate methods are shown in Table 1. Although the mapping result of the proposed AM system has a little deviation and distortion compared with Method 2, but the map is built online and the computation is much less than Method 2.

**Figure 7:**
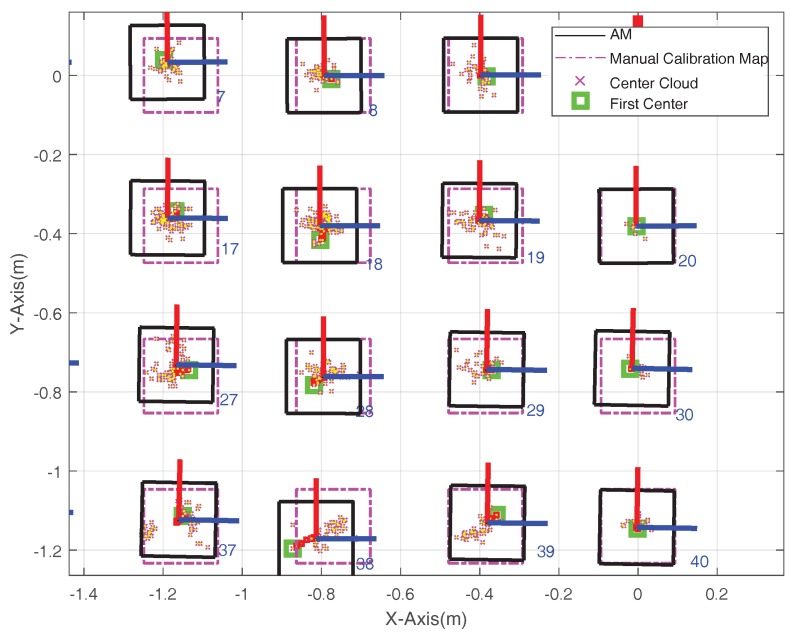
Local Enlarged Results of Figure 6d.

**Figure 8:**
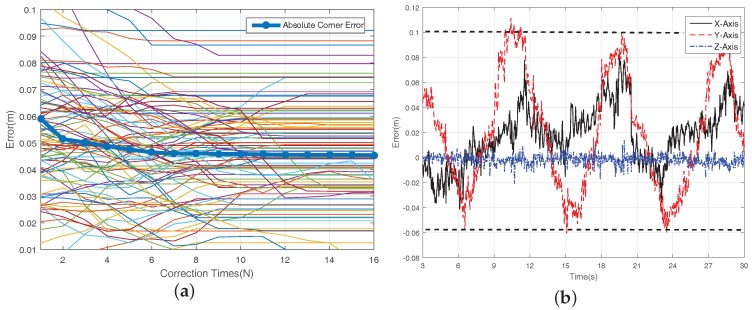
Mapping Error of Experiment Site. (**a**) Mapping Error; (**b**) Positioning Error.

The local enlarged results of markers 17–20, 27–30 and 37–40 in Figure 6d are given in Figure 7 for clear view. In Figure 8, the pink dotted square is the real position of each marker, the green bold square is the first center of each marker, the pink cross is the scattered point cloud of each marker and the black solid square is the final mapping result. It can be seen that most of the markers’ positions are closer to the real position rather than its first center, so it can be concluded that the proposed algorithm is effective.

In Figure 8a, the thin lines in the background is the corner location error of each marker, and the bold line with circle mark is the absolute corner error of the AM system which decreases with the correction times and converges to 5 cm after 12 corrections. It can be concluded that the proposed algorithm can build the map effectively and avoid the map distortion caused by the abnormal detection. Besides, the positioning error of the camera trajectory is shown in Figure 8b. The maximum error between camera estimation is 10 cm compared with the benchmark, which is satisfactory for the low cost indoor localization application. A video of the online mapping process can be seen at https://youtu.be/j6ZTGQrOdO0.

### 3.2. Experiment Results of Multi-Sensor Information Fusion

**Figure 9:**
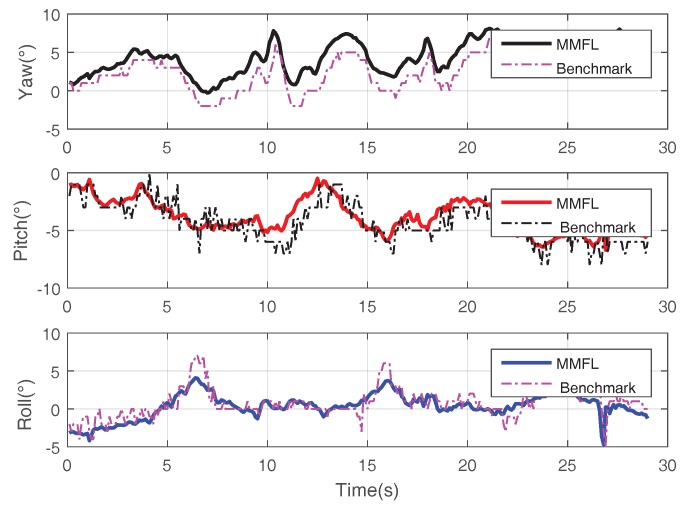
Attitude Estimation Result.

**Figure 10:**
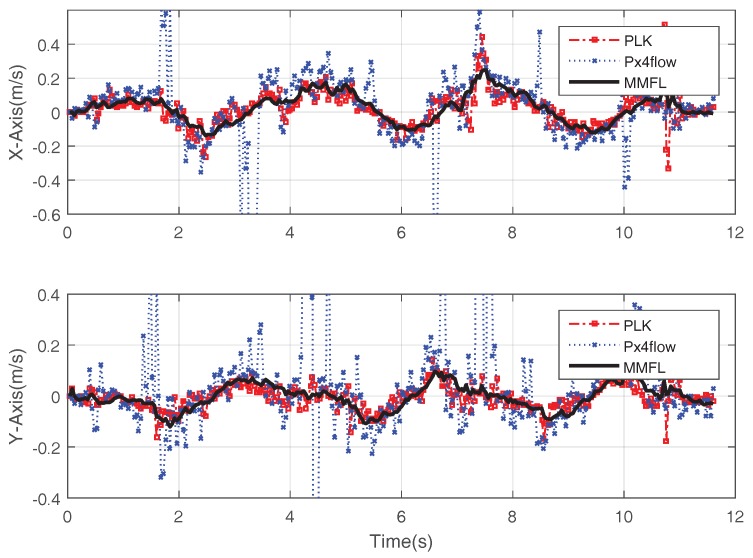
Speed Fusion Result.

**Figure 11:**
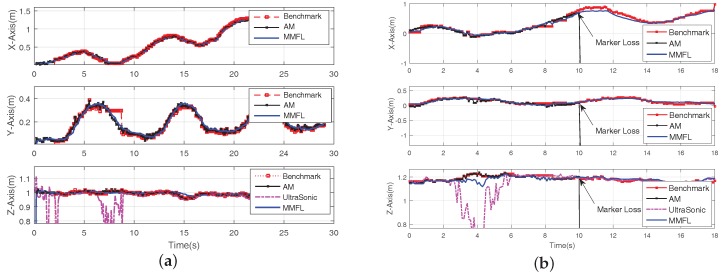
Position Fusion Result. (**a**) Without Marker Loss; (**b**) Marker Loss After the Arrowhead.

The proposed system utilizes the EKF algorithm to estimate the attitude. Figure 9 shows the attitude estimation result during one hovering experiment. The solid line in Figure 9 is the result of the proposed MMFL system and the dotted line is the calculated result based on the manual calibrated map. When the marker array is regularly arranged, the benchmark attitude is the average of all detected markers’ results with the manual calibrated map. It is clear that the result of the solid line is smoother than that of the dotted line, the small gap in Yaw is caused by the orientation error of the first detected marker and the map distortion during online mapping. In addition, the proposed system assumes that all the markers are located on the ground, thus the errors of Pitch and Roll are avoided via the static calibration. Figure 10 is the speed fusion result. The blue point line with cross is the speed measured by Px4flow. The red point line with square is the speed measured by the PLK method. The solid line is the speed estimated by the proposed MMFL system. As we can see, the speed estimation of the MMFL system is smoother and the noise of solid line is much smaller than the others. In Figure 11a, the red point line with square is the camera position calculated by the manual calibration map, the black solid line with cross is the position calculated by ArUco Mapping and the solid line is the estimation result of the MMFL system. As we can see, the result of the MMFL system is considered to be a good estimation. Meanwhile, it can be seen that the interfered ultrasonic data (dotted line) does not affect the final estimation result of the MMFL system, which shows the federated filter has good anti-interference ability. Figure 11b gives the position fusion results of the MMFL system in the long-term loss of markers. The visual location information of markers is lost after the arrowhead. As can be observed from Figure 11b, the position fusion results in *X*-Axis and *Y*-Axis have the negligible drifting, for the optical flow can still give the speed measurement. Meanwhile, the estimation of *Z*-Axis also can be corrected with the measurement of ultrasonic after the marker lost.

### 3.3. Comparison Experiment with ORB-SLAM2 System

**Figure 12:**
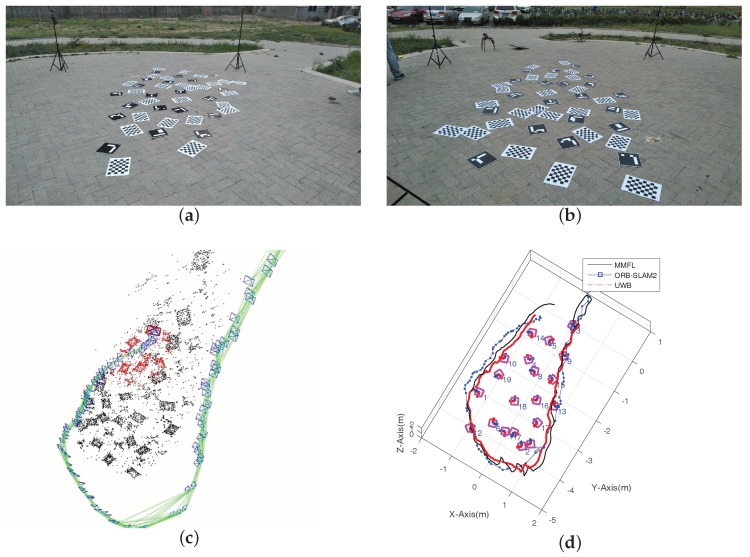
Results of Comparison Experiments with ORB-SLAM2. (**a**) The Front View of Experimental Site; (**b**) The Back View of Experimental Site; (**c**) 3D Reconstruction Result of ORB-SLAM2; (**d**) Estimated Camera Trajectory of Two Systems.

In this section, the location performance of the proposed MMFL system is compared with the ORB-SLAM2 [34], where the benchmark trajectory is provided by the localization system including four UWB base stations. The 20 markers are distributed sparsely and randomly in the 5 × 3 m region which is shown in Figure 12a,b. The MMFL system is used directly to map the experiment area, and the video recorded by the Pi camera is provided to ORB-SLAM2 for 3D reconstruction in monocular mode. The 3D reconstruction result of ORB-SLAM2 is given in Figure 12c and the estimated camera trajectory of the two candidate systems are given in Figure 12d.

As can be observed form Figure 12d, the proposed MMFL system can successfully build the map of randomly and sparsely distributed markers in featureless outdoor environment. The localization result of ORB-SLAM2 is discontinuous, which is affected by the poor feature and sparse markers. The localization performance of the MMFL system is still satisfactory compared with the benchmark provided by UWB. In addition, the data refresh rate of the ORB-SLAM2 is 17 Hz (use the same hardware platform as the Method 2 mentioned in Section 3.1) which is much lower than the proposed MMFL system (100 Hz). Compared with the results in Section 3.1, it can be concluded that a dense marker array will provide higher accuracy, which is much more suitable for MAV localization with high precision requirement.

### 3.4. Experiment Results of Hovering and Flying

**Figure 13:**
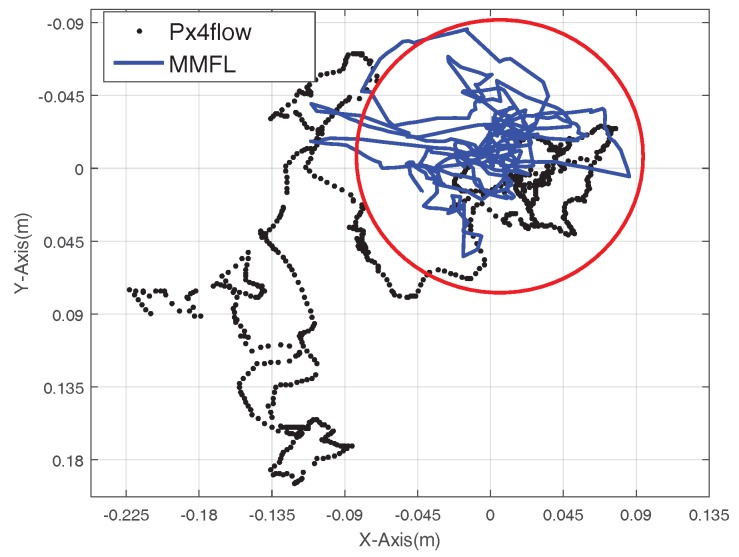
Hover Trajectory of the Proposed System and Px4Flow.

In order to test the location performance of the proposed MMFL system, the Px4flow sensor is chosen here for comparison. Firstly, a hover experiment with the Px4flow and the proposed MMFL system is implemented. The speed measurements is used as the feedback only since the Px4flow can only give the speed measurement of MAV, and the same auto disturbances rejection controller (ADRC) [35] is used in this experiment. The trajectory of one minute hovering is given in Figure 13. In Figure 13, the dotted line is trajectory with Px4flow sensor as speed feedback, which has an obvious drift. The solid line is the trajectory with the MMFL system as speed feedback, which concentrates in the circle of the 18 cm.

**Figure 14:**
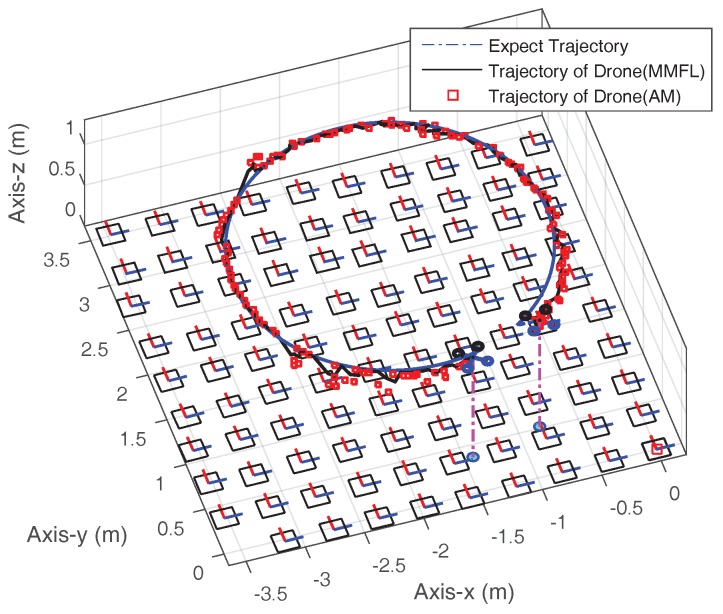
Trajectory of Autonomous Flight.

Next, two autonomous flight missions are tested. Firstly, the MAV is commanded to follow an expected trajectory, and a video of round-trip in four way points can be seen at https://youtu.be/5BbyORJeGgk. In addition, the experiment result of following a circle trajectory is shown in Figure 14. In Figure 14, the dotted line is the expected circle trajectory and the square is the original camera position calculated by ArUco Mapping, which is discontinuous. The solid line is the trajectory estimated by the MMFL system, which is smooth and can still work even if the marker is not available to be detected. It is shown that the MAV is capable of flying autonomously and following the expected trajectory based on the proposed system, and a more appropriate control parameter may provide a better result. The video of following the circle trajectory can be seen at https://youtu.be/68FB3HK49-w.

## 4. Conclusions

A multi-sensor fusion indoor location system based on ArUco marker is proposed and designed in this paper. The ArUco Mapping algorithm uses the visual information from the markers on the ground (at unknown positions) to build a map automatically. The proposed multi-sensor fusion framework utilizes the federated Kalman filter to synthesize the multi-source information from markers, inertial sensor, ultrasonic and optical flow, which can obtain continuous localization and avoid the problem of dead zone in pure marker localization. The experiment results show that our system is capable of realizing MAV localization in a 4 × 4 m marker array with centimeter level positioning and mapping accuracy. However, the proposed system is sensitive to the accumulated error in ArUco Mapping. This limitation brings the requirement of introducing the map optimize and closure loop algorithm, which is the subject to be studied in the future work. 

## Figures and Tables

**Table 1:** Performance Comparison of the Three Candidate Systems.

Index	AM Proposed in This Paper	Method 1	Method 2
ACE	0.047 m	0.36 m	0.035 m
ATE	0.085 m	0.45 m	0.061 m
